# Effect of Static and Dynamic Stretching on the Diurnal Variations of Jump Performance in Soccer Players

**DOI:** 10.1371/journal.pone.0070534

**Published:** 2013-08-05

**Authors:** Hamdi Chtourou, Asma Aloui, Omar Hammouda, Anis Chaouachi, Karim Chamari, Nizar Souissi

**Affiliations:** 1 Research Laboratory “Sport Performance Optimization” National Center of Medicine and Sciences in Sport (CNMSS), Tunis, Tunisia; 2 Research Unit (EM2S), High Institute of Sport and Physical Education, Sfax University, Sfax, Tunisia; 3 Research and Education Center, Aspetar, Qatar Orthopedic and Sports Medicine Hospital, Doha, Qatar; 4 High Institute of Sport and Physical Education, Ksar-Saïd, Manouba University, Manouba, Tunisia; Universidad Europea de Madrid, Spain

## Abstract

**Purpose:**

The present study addressed the lack of data on the effect of different types of stretching on diurnal variations in vertical jump height - i.e., squat-jump (SJ) and countermovement-jump (CMJ). We hypothesized that dynamic stretching could affect the diurnal variations of jump height by producing a greater increase in short-term maximal performance in the morning than the evening through increasing core temperature at this time-of-day.

**Methods:**

Twenty male soccer players (age, 18.6±1.3 yrs; height, 174.6±3.8 cm; body-mass, 71.1±8.6 kg; mean ± SD) completed the SJ and CMJ tests either after static stretching, dynamic stretching or no-stretching protocols at two times of day, 07:00 h and 17:00 h, with a minimum of 48 hours between testing sessions. One minute after warming-up for 5 minutes by light jogging and performing one of the three stretching protocols (i.e., static stretching, dynamic stretching or no-stretching) for 8 minutes, each subject completed the SJ and CMJ tests. Jumping heights were recorded and analyzed using a two-way analysis of variance with repeated measures (3 [stretching]×2 [time-of-day]).

**Results:**

The SJ and CMJ heights were significantly higher at 17:00 than 07:00 h (p<0.01) after the no-stretching protocol. These daily variations disappeared (i.e., the diurnal gain decreased from 4.2±2.81% (p<0.01) to 1.81±4.39% (not-significant) for SJ and from 3.99±3.43% (p<0.01) to 1.51±3.83% (not-significant) for CMJ) after dynamic stretching due to greater increases in SJ and CMJ heights in the morning than the evening (8.4±6.36% *vs.* 4.4±2.64%, p<0.05 for SJ and 10.61±5.49% *vs.* 6.03±3.14%, p<0.05 for CMJ). However, no significant effect of static stretching on the diurnal variations of SJ and CMJ heights was observed.

**Conclusion:**

Dynamic stretching affects the typical diurnal variations of SJ and CMJ and helps to counteract the lower morning values in vertical jump height.

## Introduction

Stretching is considered an essential component of an athlete’s warm-up [1]. However, most studies concerning the effect of static stretching on vertical jump performance have been inconclusive, and often shown a significant negative effect [1]–[6]. By contrast, dynamic stretching has been demonstrated to have a significant positive effect [4], [7]–[9] or, at least, no adverse effect [10]–[13] on subsequent vertical jump performance.

Maximal short-term performance in vertical jump fluctuates with time-of-day, with morning nadirs and afternoon maximum values in adults [14]–[25] and youths [26]–[28]. Jump height during the squat jump (SJ) and the countermovement jump (CMJ) has been found to be higher in the afternoon (between 16:00 and 20:00 h) than the morning (between 06:00 and 10:00 h) [17]. However, the available scientific data provide somewhat conflicting results with regard to the origin and mechanism of such diurnal rhythms [17], [29], [30].

The question then arises if dynamic or static stretching produces similar increases in body temperature and vertical jump performance in the morning and the evening. Indeed, the increase in body and muscle temperatures may increase nerve conduction velocity, enzyme activity and muscle compliance [31]–[33]. In this context, Souissi et al. [34] showed that 15 minutes rather than 5 minutes of warm-up may increase body temperature affecting diurnal variations of muscle power during the 30-second Wingate test.

The present study addressed the lack of data on the effect of different types of stretching (dynamic and static stretching) on diurnal variations of vertical jump height. We hypothesized that dynamic stretching could affect the diurnal variation of short-term maximal performance by a greater increase in jump height in the morning than the evening through increasing core temperature at this time-of-day.

## Methods

### Subjects

Twenty male soccer players (age, 18.6±1.3 yrs; height, 174.6±3.8 cm; body-mass, 71.1±8.6 kg; mean ± SD) volunteered to participate in this study. They were affiliated with a professional club competing in the first division of the Tunisian soccer league who trained five times per week for 1.5–2 hours per session. The subjects were informed in detail about the experimental procedures and the possible risks and benefits of the project, and written informed consent was obtained from each athlete or from their parents prior to participation. Subjects were informed that they could resign from participation at any time during the study. The study was conducted according to the Declaration of Helsinki and the protocol was fully approved by the Ethic Committee of the National Center of Medicine and Sciences in Sport of Tunis (CNMSS) before the commencement of the assessments. To have a group without ‘‘extreme types’’, the subjects chosen to participate in the study were all categorized as “moderately morning type” on the basis of their answers to the Horne & Östberg’s [35] self-assessment questionnaire, which assesses morningness-eveningness.

### Experimental Design

**Figure 1:**
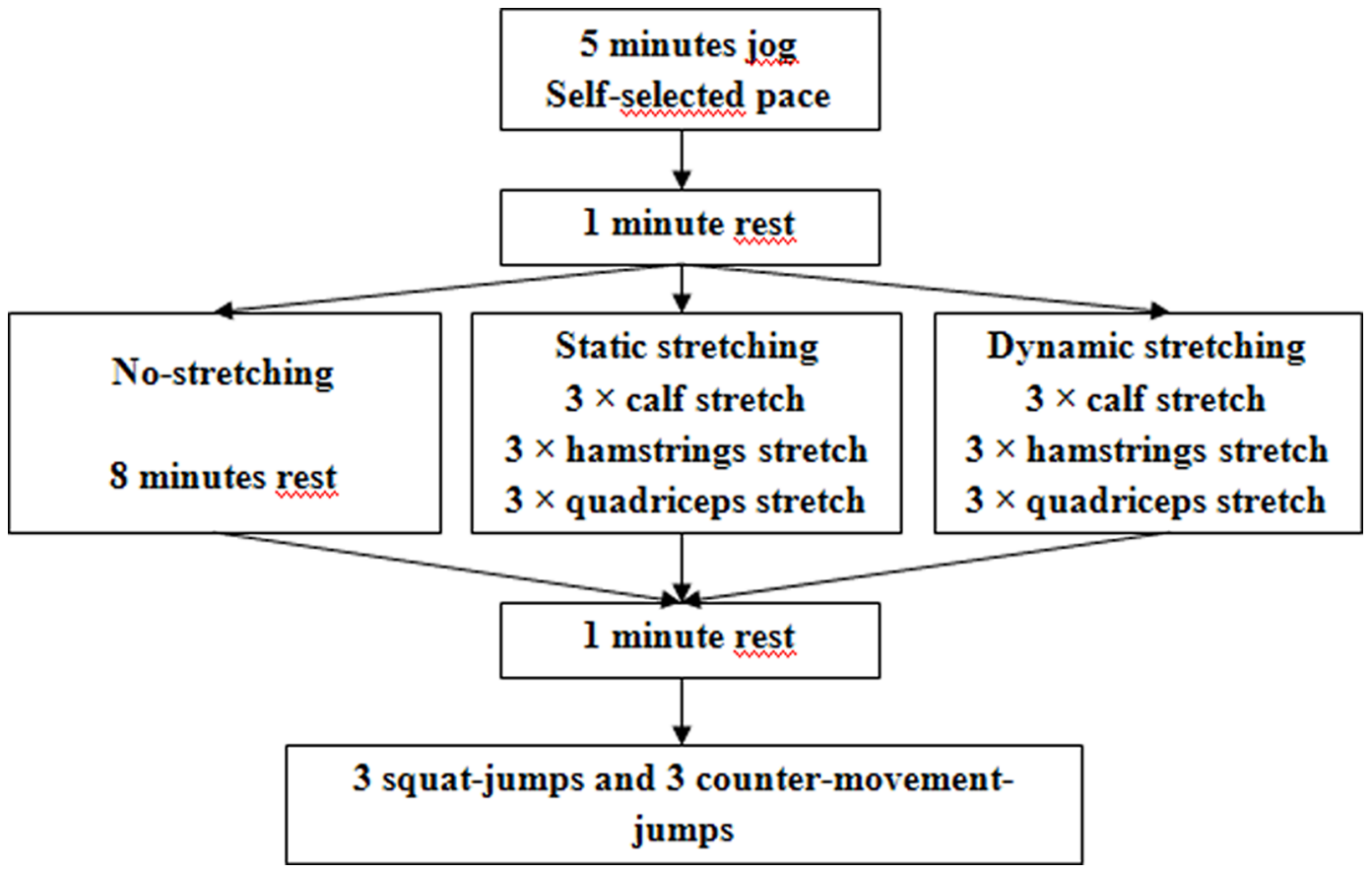
Study design.

One week before the first testing session, participants were familiarized with the vertical jumping and stretching techniques during two orientation sessions. The participants performed six experimental test sessions, with a minimum of 48 hours between testing sessions, in a random order: three in the morning (07:00 h) and three in the evening (17:00 h) with static stretching, dynamic stretching or no-stretching. One minute after completing the specific warm-up protocol (see below), the athletes performed 3 SJs and 3 CMJs with a 30 second passive recovery between each jump. The best performance (i.e., maximal jump height) was used for further analysis. The experimental procedure is summarized in Figure 1.

Oral temperature was recorded by digital clinical thermometer (Omron®, Paris, France; accuracy ±0.05°C) at the beginning of each test session after the subjects had been in a supine position for 15 minutes and following each warm-up protocol. Core temperature is considered to be a chronobiological marker, i.e., representative of the functioning in the circadian system [36]. Moreover, core temperature was measured to verify that the selected times adhere to the expected maxima and minima in anaerobic performance [37].

To minimize confounding factors, instructions related to sleep and diet were given to the subjects before the experiment, as recommended by Bougard et al. [38]. On the night preceding each test session, participants were asked to keep to their usual sleeping habits, with a minimum of 7 hours of sleep. All subjects were interviewed before each session to verify compliance with these directions. Subjects were also requested to maintain their habitual level of physical activity and to avoid strenuous activity the day before each test session [22], [39]. Before the morning test sessions, only one glass (15 to 20 cl) of water was allowed, to avoid postprandial thermogenesis effects. After the morning sessions, unrestricted food intake was allowed. Before the evening test sessions, subjects had the same standard isocaloric meal at 12:00 h, which was concluded at least 5 hours before the tests. After this meal, water was allowed *ad libitum*.

### Warm-up Protocols

Subjects performed a 5 minute self-paced general warm-up consisting of low-intensity aerobic exercise followed by 8 minutes of designated stretching (specific warm-up) protocol for the static stretching and dynamic stretching test sessions or 8 minutes of rest for the no-stretching control condition. The time between finishing the general warm-up and beginning the stretching protocol was approximately 1 minute.

### Static and Dynamic Stretching

The static stretching and the dynamic stretching consisted of 3 lower-extremity exercises that were performed for 3 repetitions for 20 seconds with 7–8 second muscle release between each repetition. The muscles stretched were the hamstrings, the quadriceps and the calf muscles. For the static stretching (Table 1), each stretch was held in a position at which the participant verbally indicated that he had stretched the muscle to a point of mild discomfort [40]. For the dynamic stretching exercises (Table 2), each athlete intentionally contracted the antagonist of the target muscle and performed the dynamic movements every 2 seconds under the verbal count of the experimenter [4]. Each exercise was performed 5 times slowly and then 10 times as quickly as possible without bouncing [40].

**Table 1:** Static stretching exercises.

**Calf**	The subject remained in the supine position with the knee fully extended while the investigator dorsiflexed his ankle joint.
**Hamstrings**	The subject remained in the supine position with the knee fully extended while the investigator flexed his hip joint with the knee remaining in extended position.
**Quadriceps**	While in a lying position facing the ground, the subject’s heel touched his buttock, and then the knee was lifted up such that the hip joint was extended. The investigator fully flexed the knee joint of the subject in the prone position.

**Table 2:** Dynamic stretching exercises.

**Calf**	• The subject in a standing position, raised one foot from the floor and fully extended the knee.
	• The subject then contracted his dorsiflexors intentionally and dorsiflexed his ankle joint such that his toe was pointing upward.
**Hamstrings**	The subject in a standing position, contracted the hip flexors intentionally with the knee fully extended and flexed his hip joint such that his leg was swung up to the front.
**Quadriceps**	• The subject in a standing position, raised a foot from the floor and lightly flexed his hip joint with the knee slightly flexed.
	• The subject then contracted his hip extensors intentionally and extended his hip and knee joints such that his leg was extended backwards.

### Squat Jump (SJ) and Countermovement Jump (CMJ) Tests

Participants were asked to perform maximal vertical SJs and CMJs on an infrared jump system (Optojump, Microgate, Bolzano, Italy) interfaced with a microcomputer. The Optojump system measures flight and contact times with an accuracy of 1/1000 of a second. For the SJs, subjects got into a squat position and, after a brief pause, jumped upwards as quickly and as high as possible. No downwards motion was allowed immediately prior to jumping upwards. For the CMJs, subjects initiated the jump from an extended leg position, descended to a squat position, and then immediately performed an explosive concentric action to reach maximal height. In both tests, participants were instructed to keep their hands on the hips and to minimize lateral and horizontal displacements throughout the jumps [41].

### Statistical Analyses

Statistical tests were processed using the STATISTICA Software (StatSoft, France). Mean, and standard deviation (SD) were calculated for the selected variables. Once the assumption of normality had been confirmed by the Shapiro-Wilk *W*-test, parametric tests were used. SJ and CMJ heights were analyzed using a two-way analysis of variance (ANOVA) with repeated measures (3 [stretching]×2 [time-of-day]). The difference between the morning and the evening values (i.e., the diurnal gain) during the static stretching, dynamic stretching and no-stretching conditions was calculated and analyzed using a one-way ANOVA with repeated measures. Also, the delta-changes of performance between no-stretching and static stretching, and between no-stretching and dynamic stretching, were calculated at 07:00 and 17:00 h and analyzed using a two-way ANOVA with repeated measures (2 [stretching]×2 [time-of-day]). Oral temperature data were analyzed using a three-way ANOVA with repeated measures (3 [stretching]×2 [before/after]×2 [time-of-day]). When appropriate, significant differences between means were assessed using the Tukey *post-hoc* test. To assess the data practical significance, effect sizes were calculated as partial eta-squared, η_p_^2^. Test-retest reliability was assessed by intra-class correlation coefficients (ICCs) and standard error of measurement (SEM). Statistical significance for all analyses was set at p≤0.05.

## Results

**Figure 2:**
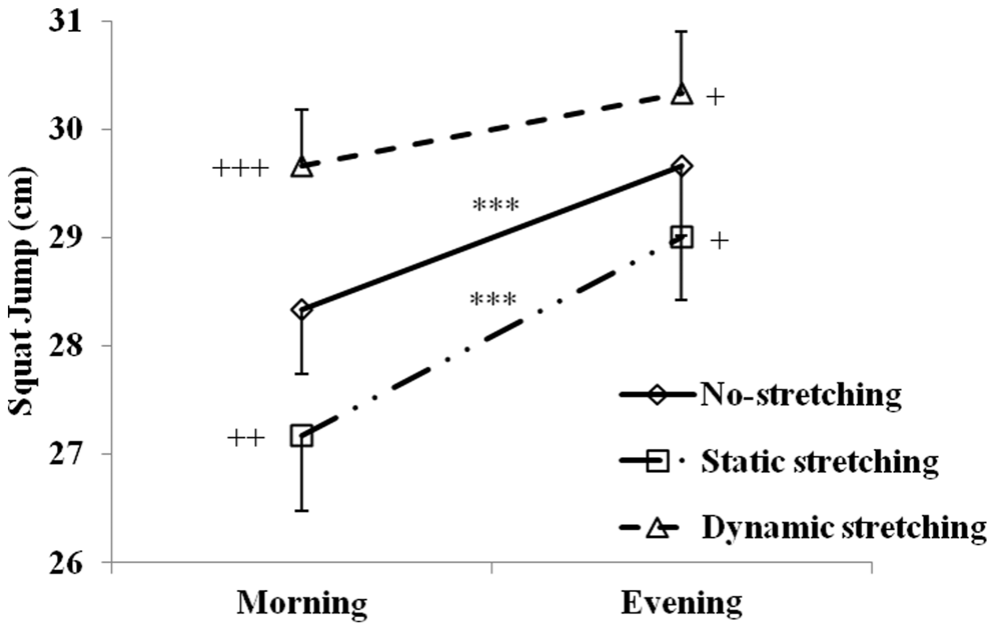
Mean ± SD for squat jump (SJ) performances recorded at 07:00 h and 17:00 h after the no-stretching, static stretching, and dynamic stretching protocols. ***: significant difference between 07:00 and 17:00 h at p<0.001. +, ++, +++: Significant differences in comparison with NS at p<0.05, p<0.01, and p<0.001 respectively.

**Figure 3:**
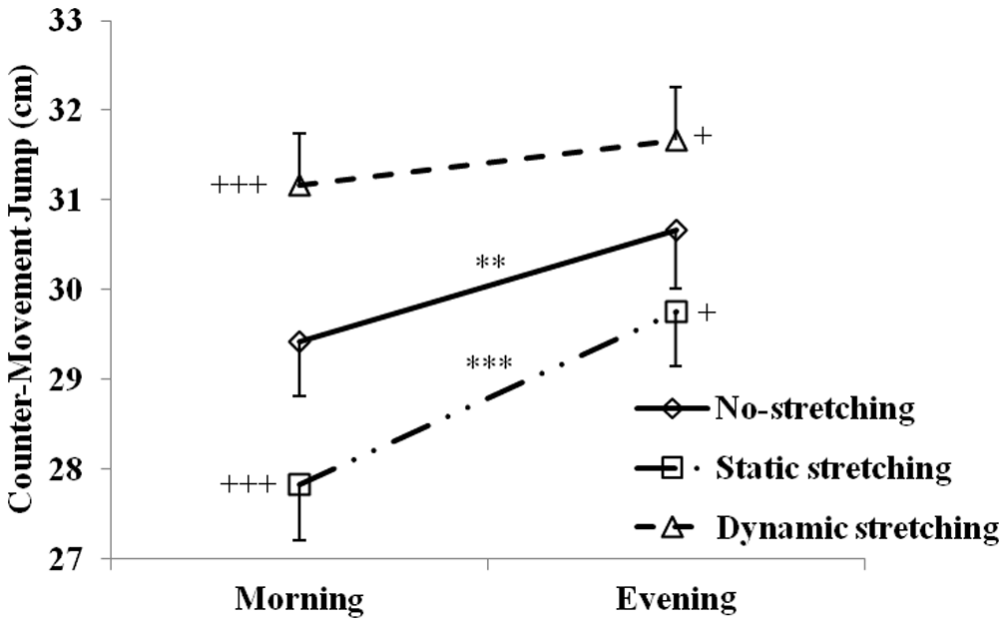
Mean ± SD for counter-movement jump (CMJ) performances recorded at 07:00 h and 17:00 h after the no-stretching, static stretching, and dynamic stretching protocols. **, ***: significant difference between 07:00 and 17:00 h at p<0.01 and p<0.001 respectively. +, +++: Significant differences in comparison with NS at p<0.05 and p<0.001 respectively.

The SJ and CMJ heights measured at 07:00 and 17:00 h after static stretching, dynamic stretching and no-stretching protocols are displayed in Figures 2 and 3.

### Squat-jump

The SJ showed a high reliability between test-retest sessions (ICC higher than 0.88 and absolute SEM lower than 1.5 cm).

There was a significant main effect for stretching (F  = 33.3, p<0.001, η_p_^2^ = 0.77) and time-of-day (F  = 34.6, p<0.001, η_p_^2^ = 0.72). Likewise, the interaction stretching×time-of-day was significant (F  = 3.6, p<0.05, η_p_^2^ = 0.59).

After the no-stretching protocol, the *post-hoc* analysis showed that SJ height was significantly higher at 17:00 than 07:00 h (p<0.01) with an amplitude of 4.2±2.81%. These diurnal variations were blunted after the dynamic stretching by an attenuated morning-evening difference (4.2±2.81% (p<0.01) *vs.* 1.81±4.39% (not-significant)) but persisted after the static stretching.

Moreover, SJ height increased after dynamic stretching (p<0.001 and p<0.05 at 07:00 and 17:00 h, respectively) and decreased after static stretching (p<0.01 and p<0.05 at 07:00 and 17:00 h, respectively) in comparison with no-stretching at 07:00 and 17:00 h. The improvement of performance after dynamic stretching compared to no-stretching was significantly higher in the morning than the evening (8.4±6.36% *vs.* 4.4±2.64%, p<0.05). However, the delta-change was not significantly different between the morning and the evening after static stretching.

### Countermovement-jump

The CMJ showed a high reliability between test-retest sessions (ICC higher than 0.94 and absolute SEM lower than 1.2 cm).

There was a significant main effect for stretching (F  = 43.5, p<0.001, η_p_^2^ = 0.81) and time-of-day (F  = 24.4, p<0.001, η_p_^2^ = 0.74). Likewise, the interaction stretching×time-of-day was significant (F  = 3.5, p<0.05, η_p_^2^ = 0.61).

The *post-hoc* test revealed that CMJ was significantly higher in the evening than the morning (p<0.01) during the no-stretching test sessions with an amplitude of 3.99±3.43%. These diurnal fluctuations persisted during the static stretching sessions but disappeared during the dynamic stretching sessions with a reduced diurnal amplitude (3.99±3.43% (p<0.01) *vs.* 1.51±3.83% (not-significant)).

Furthermore, in the morning and the evening, jump height during the CMJ increased after dynamic stretching (p<0.001 and p<0.05 at 07:00 and 17:00 h, respectively) in comparison with no-stretching, with a greater improvement at 07:00 h than 17:00 h (10.61±5.49% *vs.* 6.03±3.14%, p<0.05). However, jump heights decreased after static stretching in comparison with no-stretching at 07:00 and 17:00 h (p<0.01 and p<0.05 respectively). The delta-change was not significantly different between the morning and the evening after static stretching.

### Oral Temperature

**Figure 4:**
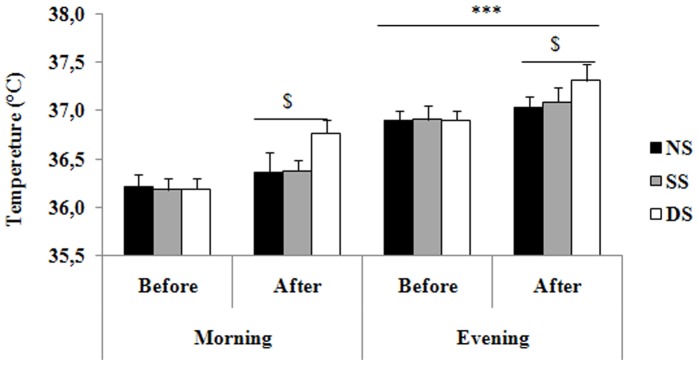
Mean ± SD for core temperature recorded at 07:00 h and 17:00 h before and after the no-stretching, static stretching, and dynamic stretching sessions. ***: significant difference between 07:00 and 17:00 h at p<0.001. $: Significant differences between before and after each stretching protocol at p<0.001.

Oral temperatures recorded before and after static stretching, dynamic stretching and no-stretching protocols are presented in Figure 4. Oral temperature increased significantly after the three conditions (p<0.001) and was higher in the evening (all p<0.001) compared to the morning both before and after each stretching condition. However, the amplitude of the diurnal variations was significantly reduced after dynamic stretching compared to no-stretching (1.92±0.39% *vs.* 1.47±0.42%, p<0.01) due to a higher increase in oral temperature in the morning than in the evening (1.56±0.36% *vs.* 1.12±0.33%, p<0.01). Moreover, compared to before the stretching protocols, the increase in oral temperature after dynamic stretching was significantly higher than after no-stretching and static stretching protocols (both p<0.01).

## Discussion

The purpose of this study was to determine whether acute static stretching and dynamic stretching had any effect on the diurnal fluctuations of vertical jumping performance. The primary finding was that dynamic stretching may reduce the amplitude of the diurnal variations of SJ and CMJ heights by producing a greater increase in short-term maximal performance in the morning compared to the evening. By contrast, static stretching did not affect the diurnal variations in SJ and CMJ heights.

During the no-stretching control condition, the results demonstrated that SJ and CMJ heights were higher in the evening than the morning. Diurnal variations have been demonstrated during continuous (e.g., Wingate test) [18]–[21], [23]–[25] and intermittent (e.g., repeated sprint ability test) [23], [42], [43] cycling exercises, or short-term maximal performances (e.g., maximal voluntary contraction, SJ, CMJ, etc.) [18], [20]. However, the underlying mechanisms of these diurnal fluctuations are still debated [44], [45]. Some authors have hypothesized a causal link between the diurnal fluctuations of core temperature and short-term maximal performances [17], [37], [42], [46]. Indeed, the increase in body temperature in the evening hours may increase the conduction velocity of action potentials, and this could result in better motor coordination and so produce higher muscle power output at this time-of-day [15]. Moreover, by examining the electromyographic (EMG) activities of the lower limbs during the Wingate test, previous study found that the diurnal variations in muscle power and fatigue were likely to be linked to conditions prevailing at the peripheral rather than central level [25].

Concerning the effects of static stretching, the present results showed that jumping heights during the SJ and CMJ tests were impaired in the morning and in the evening. Although some previous studies had been unable to show any adverse effect of the static stretching [11], [47]–[49], others showed that static stretching may adversely affect short-term maximal performance [1]–[6]. The discrepancies between the results of the present study and those in the literature could be attributed to the age and training status of the subjects, stretch duration, intensity and volume, and to other factors (for further details, see [31]). Robbins and Scheuermann [50] investigated the effects of 3 different volumes of static stretching on vertical jump height and showed that the greatest stretching volume, i.e., 6 sets of 15 seconds, decreased jump performance by an average of 1.9 cm. The exact mechanism for the decline in performance induced by static stretching is not fully understood; however, authors have speculated that a decrease in muscle activation and musculo-tendinous stiffness could be the main causes [2]. Nelson et al. [51] suggested that increased muscular compliance as a result of stretching might mean that the muscle will go through a greater period of unloaded shortening before taking up sufficient slack to then be able to transfer generated force to the bone. Consequently, cross-bridges may be at a less-than-optimal length much sooner in the movement. Moreover, Hough et al. [4] suggested that the static stretching may cause some neurological impairment that result in decreased muscle activation. This suggestion is supported by previous research demonstrating reductions in EMG activity of the quadriceps [52] and triceps surae [1] muscles after static stretching. Decreased muscle activation after static stretching could be explained by a number of factors including the Golgi tendon reflex and the mechanoreceptor (type III afferent) and nociceptor pain feedback (type IV afferent) responses [53]. As in the present study, the static stretching was assisted; this may activate the Golgi tendon organs that would cause a reciprocal inhibition of the motor neurons of the antagonist muscle, thereby causing autogenic inhibition [4].

Although some previous studies reported that dynamic stretching did not improve short-term explosive performance [8], [11], [12], [54], others showed facilitation of jump performance [4], [7]–[9]. Behm and Chaouachi [31] concluded that dynamic stretching routines are a preferred method for improving explosive muscle contractions. In the present study, the improvements in jump height during the SJ and the CMJ were greater in the morning than the evening. However, to the authors’ knowledge, no previous study has compared the effects of static and dynamic stretching on the diurnal variations of short-term explosive muscle performance. Therefore, it is not possible to compare the present results with those of others. The mechanisms by which dynamic stretching improves muscular performance have been suggested to be elevated muscle and body temperature [2]. The greater increase in core temperature after dynamic stretching in the morning compared with the evening may induce faster and more forceful muscle contractions in the morning. Indeed, the higher body temperature may enhance metabolic reactions, increase the extensibility of connective tissue, reduce muscle viscosity, and increase the nerve conduction velocity of action potentials [15], [55], [56]. Moreover, Hough et al. [4] showed that EMG activity during a vertical jump exercise increased after dynamic stretching. This may reflect an increased recruitment of active neurons supplying each motor unit and so facilitate motor unit activation through neuromuscular propagation. Previous studies showed a higher EMG activity during the 30 second Wingate test in the evening compared to the morning [25], [27]. Thus, dynamic stretching performed in the morning hours could enhance muscle EMG activity and, therefore, reduce the amplitude of the diurnal variation of short-term maximal performance.

Limitations of this study include the fact that all participants were male soccer players. Future investigations should focus on determining any modification to diurnal variations of short-term maximal performance in females. Moreover, short-term maximal performance was tested using only the SJ and CMJ. Therefore, acute effects of stretching on the daily fluctuations of upper body performance should also be investigated. Since the 3 CMJ repetitions were performed after the 3 SJs over a time span of only about 3 minutes, the conclusions from the present study are to be interpreted as an effect not only upon single short-term explosive power efforts but also, to some extent, upon powerful and repeated short bursts of exercise. For more accurate data regarding this issue, a similar protocol could be performed using repeated sprint ability (RSA) – investigating single power, mean power and fatigue index variables and alterations in these with time-of day and after static and dynamic stretching.

### Conclusion

The results of the present study confirm that (i) static stretching is detrimental and (ii) dynamic stretching is beneficial and can overcome decreases in jump height in the morning. Thus, athletes required to compete in the morning hours and to produce powerful efforts may be advised to include dynamic stretching exercises in their pre-competition warm-ups.
